# How Do Price Minimizing Behaviors Impact Smoking Cessation? Findings from the International Tobacco Control (ITC) Four Country Survey

**DOI:** 10.3390/ijerph8051671

**Published:** 2011-05-20

**Authors:** Andrea S. Licht, Andrew J. Hyland, Richard J. O’Connor, Frank J. Chaloupka, Ron Borland, Geoffrey T. Fong, Nigar Nargis, K. Michael Cummings

**Affiliations:** 1 Department of Health Behavior, Roswell Park Cancer Institute, Elm and Carlton Streets, Buffalo, NY 14263, USA; E-Mails: andrew.hyland@roswellpark.org (A.J.H.); richard.o'connor@roswellpark.org (R.J.O.); michael.cummings@roswellpark.org (K.M.C.); 2 Department of Social and Preventive Medicine, University at Buffalo, Buffalo, NY 14214, USA; 3 Department of Economics, University of Illinois at Chicago, Chicago, Illinois 60607, USA; E-Mail: fjc@uic.edu; 4 Vic Health Center for Tobacco Control, the Cancer Council Victoria, Carlton, VIC 3053, Australia; E-Mail: ron.borland@cancervic.org.au; 5 Department of Psychology, University of Waterloo, Waterloo, Ontario N2L 3G1, Canada; E-Mail: gfong@watarts.uwaterloo.ca; 6 Department of Economics, University of Dhaka, Dhaka-1000, Bangladesh; E-Mail: nnargis2100@gmail.com

**Keywords:** tobacco, cessation, price, tax, policy, socio-economic status

## Abstract

This paper examines how price minimizing behaviors impact efforts to stop smoking. Data on 4,988 participants from the International Tobacco Control Policy Evaluation (ITC) Four-Country Survey who were smokers at baseline (wave 5) and interviewed at a 1 year follow-up were used. We examined whether price minimizing behaviors at baseline predicted: (1) cessation, (2) quit attempts, and (3) successful quit attempts at one year follow up using multivariate logistic regression modeling. A subset analysis included 3,387 participants who were current smokers at waves 5 and 6 and were followed through wave 7 to explore effects of changing purchase patterns on cessation. Statistical tests for interaction were performed to examine the joint effect of SES and price/tax avoidance behaviors on cessation outcomes. Smokers who engaged in any price/tax avoidance behaviors were 28% less likely to report cessation. Persons using low/untaxed sources were less likely to quit at follow up, those purchasing cartons were less likely to make quit attempts and quit, and those using discount cigarettes were less likely to succeed, conditional on making attempts. Respondents who utilized multiple behaviors simultaneously were less likely to make quit attempts and to succeed. SES did not modify the effects of price minimizing behaviors on cessation outcomes. The data from this paper indicate that the availability of lower priced cigarette alternatives may attenuate public health efforts aimed at to reduce reducing smoking prevalence through price and tax increases among all SES groups.

## 1. Introduction

Raising cigarette prices has been shown to be an effective way to control smoking [1–5], as past literature has demonstrated that higher cigarette prices result in decreased cigarette consumption, increased quit attempts, and higher rates of smoking cessation [4–8]. Among US adults, a 10% increase in price is estimated to result in a 3–5% decrease in cigarette demand, while most estimates center around a 4% reduction [3,9–12]. Price elasticity estimates for Western European countries are similar to those of the US and other high income countries [13]. In Canada widespread smuggling problems can make price elasticity calculations difficult; however, after adjusting for smuggling, estimates are close to those in other high-income countries again centering on a 4% reduction in demand for a 10% increase in price [14]. Tax-induced cigarette price increases may represent a key policy option to drive cessation as evidence suggest they are effective in reducing smoking prevalence and result in large gains in both total and quality adjusted life years [15].

According to the Centers for Disease Control and Prevention, smokers with lower incomes, those from minority populations, and those who are younger are more likely to reduce the number of cigarettes smoked per day or quit in response to a price increase [16]. In addition to econometric evidence, other literature also supports the hypothesis that higher prices result in decreased consumption. For example, after a tax increase in Massachusetts [17], there was a decline in the number of packs of cigarettes taxed per capita sold. Immediately following a tax increase in California [18], a significantly greater proportion of smokers reported making quit attempts. Additionally, after intense tobacco control measures were enacted in New York City in 2002, the smoking prevalence decreased 11% largely due to the rapid and large increase in both state and city excise taxes [4].

The interaction between socio-economic status (SES) and response to tobacco control policies represents a potentially important avenue to reduce disparities in tobacco use. A review addressing the social inequalities in smoking for various tobacco control policies published by the Centre for Review and Dissemination (University of York, 2008) found strong evidence of a negative social gradient between income and the price of tobacco products. This indicates that minority or disadvantaged groups may be more responsive to cigarette price increases compared to more advantaged strata [19]. There is suggestive evidence of a negative social gradient by occupation as well, but this association was limited to the United Kingdom [19]. Additionally, the forthcoming IARC handbook also provides evidence that socially disadvantaged adults are more price sensitive [13].

**Figure 1.**
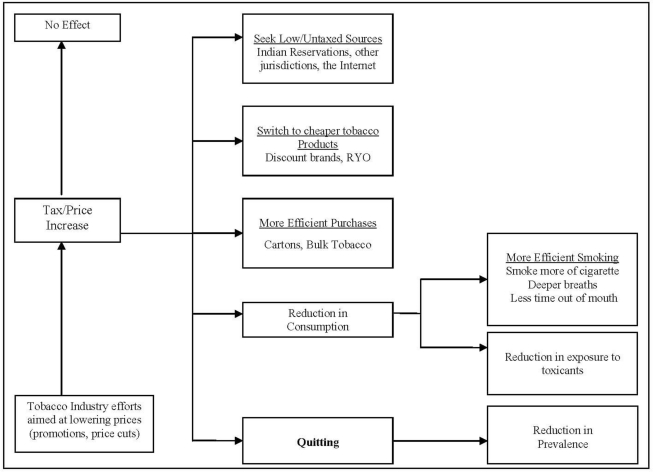
Modified Compensatory Model of Price effects in response to a price or tax increase.

In addition to the intended cessation outcomes associated with price or tax increases, smokers also have several options available to minimize their expenditures for cigarettes (see Figure 1). Based on a compensatory model of price effects developed by investigators on the International Tobacco Control Policy Evaluation Project [20], unintended outcomes associated with a price increase are possible, including: (1) having no effect on the smokers’ behavior, (2) cutting back on cigarettes smoked, or (3) engaging in techniques to help alleviate the price burden. Previous literature has suggested that at least half of the decrease in cigarette demand occurs as a result of reductions in individual cigarette consumption, as opposed to complete cessation [1,7]. Although some evidence suggests that consumers have become more price sensitive over time [5,14], a recent IARC review has concluded that cigarette price elasticity has remained relatively stable over time and across different price levels in high income countries [13].

Econometric evidence of cigarette price and cessation consistently reports an inverse relationship between higher prices and cessation [3,9–14]. However, the effect on cessation behaviors with use of other compensatory behaviors has been somewhat limited. Based on previous literature, smokers faced with higher cigarette prices may seek out cheaper tobacco outlets [6,21,22], may find tax-free or tax-reduced sources [4,6,21], may shift to lower cost forms of tobacco such as cheaper brands or roll-your-own tobacco [6,21,22], or they may choose to purchase tobacco products in bulk or in cartons [21,22]. Previous literature using data from the International Tobacco Control (ITC) study did not find an overall effect of reduced cessation with use of low or untaxed sources. However, those who used these sources were approximately 30% less likely to report making quit attempts [7]. A study from the United States found that use of discount brands was associated with about a 20% reduction in cessation at follow-up [23]. However, these studies have not assessed the use of price minimizing behaviors and the effect on cessation by individuals in various socio-economic groups. Given the known relationship between SES and smoking, the relationship between SES and use of price minimizing behaviors [24], and the differences in price responsiveness of lower SES smokers, SES may be an important modifier on the impact of price minimizing behaviors and cessation outcomes.

Access to cheaper tobacco sources, such as discount/generic brands, low/untaxed sources, or bulk purchasing, can undermine the effects of price increases because they allow for alternative options instead of quitting or cutting down. The possible modifying role of SES on this relationship is important, but few studies have examined socio-economic differences in compensatory behaviors aimed at alleviating the burden of increasing cigarette prices and their impact on smoking cessation outcomes. This information may be especially useful in directing future public health policies. This paper examines how price minimizing behaviors impact efforts to stop smoking and remain off cigarettes. Additionally, it assesses how SES modifies the relationship between use of lower priced tobacco products and cessation behaviors.

## 2. Experimental Section

### 2.1. Data Source

Data analyzed from this study come from several survey waves of the International Tobacco Control (ITC) Policy Evaluation Project-Four Country Survey (ITC-4 Survey). The conceptual framework and methodology of this project have been published in full elsewhere [25,26]. Briefly, the objective of the ITC-4 Project is to evaluate the psychosocial and behavioral effects of the goals and interventions set forth in the Framework Convention on Tobacco Control (FCTC) [25]. The ITC Study has both cross-sectional and longitudinal study arms. Following a “quasi-experimental” study design, researchers can evaluate the effectiveness of tobacco control policies by following and comparing both between and within country trends occurring after implementation [25].

The ITC-4 survey consists of parallel prospective surveys which follow a nationally representative cohort of adult smokers in the four largest English-speaking countries: the United States (US), Canada (CA), the United Kingdom (UK), and Australia (AU). Participants were identified using stratified random digit dialing and interviews were conducted using computer assisted telephone interview (CATI) software at multiple research facilities. A complex sampling design, incorporating population stratification, unequal probabilities of selection, and random digit dialing techniques, is used to gain a representative sample from each country [26]. Interviews were conducted in both English and French, depending on the primary language of the participant. Strict protocols were followed to ensure methodological congruity across research facilities and between the two languages [25,26].

A strength of the ITC-4 project is the longitudinal study design. However, this design is also subject to attrition over time. To compensate for respondents who are lost to follow up at each survey wave, lost cohort members are replenished by newly recruited respondents following similar recruitment strategies as the original cohort. Replenishment is done to ensure at least 2,000 participants from each country are present at each survey wave. Data are weighted to adjust for the changing demographic characteristics over time due to the replenishments of the cohort [26].

### 2.2. Study Populations

A total of 7,038 smokers were interviewed as part of the wave 5 data collection in ITC. Study participants included in this analysis are smokers at baseline who were re-contacted one year later at the wave 6 interview (n = 4,988). Wave 5 data were collected from October 2006 until February, 2007. Wave 6 data collection began in September 2007 and was completed in February 2008. Among the participants included in this study population, 1,245 (25.0%) were in Canada, 1,130 (22.7%) were in the US, 1,263 (25.3%) were in the UK, and 1,350 (27.1%) were in Australia. In the current study, baseline demographic characteristics between smokers who were retained in the cohort and those who were lost to follow-up at wave 6 were generally similar, suggesting random loss to follow-up among this study population. The demographic and behavioral characteristics of this study population are described in Table 1.

A second set of longitudinal analyses encompasses the time period from October, 2006 until July, 2009 and utilizes waves 5, 6, and 7 of the ITC-4 study. This analysis was completed among 3,387 respondents who were smokers at waves 5 and 6 and were followed through to wave 7. Demographic characteristics in this sub-population were similar to those in the wave 5-wave 6 longitudinal cohort.

### 2.3. Definition of Purchasing Behaviors

Identification of last purchase from a low or untaxed venue was determined from each respondents’ last reported purchase location. Responses were categorized as follows: (1) Convenience store, gas station, newsstand; (2) Grocery store, discount/”big box” outlet stores; (3) Discount tobacco outlet venues or tobacco specialty shops; (4) entertainment venues such as bars, restaurants, casinos; (5) liquor stores; (6) from a vending machine; (7) Military commissaries; (8) Duty-free shops; (9) Indian Reservations; (10) Outside the state/country of residence; (11) by Internet or telephone; or (12) from a friend, relative, or other independent seller. Purchases last made from: military commissaries (US only), Indian Reservations (US and CA only), duty free shop, outside the state or country, by telephone, Internet, someone else, or a friend or relative were included as “low/untaxed purchases” for the construct. All other sources were considered to be from full taxed venues.

For each respondent, the specific brand and variety of tobacco last purchased was reported and was used as a proxy measure of recent brand and variety exposure. Varieties and/or pack descriptors were combined into brand families which were further categorized as being premium brands, discount brands, or “roll-your-own” (RYO) tobacco varieties based on previous work [27] and current cigarette market research.

Current smokers who reported purchasing factory made cigarettes at last purchase were queried on the unit of tobacco purchased (carton, pack, or loose/single cigarettes). Based on this variable, a measure of price avoidance by purchasing tobacco in cartons was constructed. Respondents purchasing packs or single/loose cigarettes were categorized as non-participants, while respondents who purchased tobacco in a carton at last purchase were considered to be participating in this price avoidance technique. Respondents who purchased RYO tobacco at last purchase were excluded from this construct.

A composite construct was derived to assess whether use of at least one price or tax avoidance behavior at baseline was associated with cessation outcomes at follow-up. Respondents were given a score of “1” if they reported using any of the above price/tax avoidance behaviors at baseline. Individual scores were added together to obtain a measure of any price and tax avoidance at last purchase. A maximum score of 3 could be obtained for smokers of factory-made cigarettes; however, a maximum score of 2 could be assigned to RYO tobacco users due to the inability to purchase RYO tobacco in cartons. This price and tax avoidance score was categorized into “no use” (score = 0) *vs.* “any use” (score ≥ 1) at last purchase.

Additionally, “any use” was further categorized in which respondents with a score of ≥2 were categorized as participating in multiple price minimizing behaviors simultaneously while respondents with a score = 1 were considered to be participating in minimal price and tax avoidance. These measures of “any price/tax avoidance” and “varying price/tax avoidance” were assessed as predictors of cessation outcomes at follow-up.

The preceding measures of price minimizing behaviors are proxies for purchasing tobacco products at a reduced cost. However, from these behaviors alone it is not clear whether lower price paid per cigarette actually results in reduced cessation indicators. A measure of “price per cigarette” can be constructed using individual data concerning the unit of tobacco purchased (carton, pack, or loose), the number of cartons/packs purchased, and the number of cigarettes per pack. This measure was not calculated for “roll your own” users because a valid measure of cigarettes per package of loose tobacco is not feasible. All price per cigarette values were converted to $USD (U.S. Dollars) for the year 2006. Outliers, defined as price measures outside of 3 Standard Deviations of the mean were excluded.

### 2.4. Changes in Location of Last Purchase between Waves 5 and 6

Changing purchase location (full tax *vs.* low/untaxed venue) among smokers in the baseline and first follow-up interview (waves 5 and 6) was used as a predictor cessation (at wave 7) following long term use of this price minimizing behavior. Differences in purchase location for current smokers at waves 5 and 6 were categorized as follows: (1) full tax source at both waves; (2) full tax source at wave 5, low/untaxed source at wave 6; (3) low/untaxed source at wave 5, full tax source at wave 6; and (4) low/untaxed source at both survey waves. Outcomes assessed include making quit attempts and cessation between waves 6 and 7.

### 2.5. Covariates

Other covariates included in all multivariate analyses were country of residence (Canada, the United States, the United Kingdom, and Australia), age at time of interview (18–39, 40–54, 55+), sex (female, male), minority status (white, English speaking *vs.* minority defined as non-white, or non-English speaking in Australia), and Heaviness of Smoking Index (composite of cigarettes per day and time to first cigarette after waking; respondent assigned a number ranging from 0 to 6, with 6 relating to a heavier addiction).

### 2.6. SES Composite Variable

In this study, a composite SES variable was created by combining each respondent’s educational attainment and annual household income. Educational attainment varied by country due to different education systems. A derived measure of educational attainment was created which categorized respondents into low (completed high school or less), moderate (training, technical school, some university) or high education (university degree or higher) while taking into account varying educational systems between the four countries. Average annual household income was defined as the total income before taxes for all persons in the household combined. A derived measure of household income was categorized into three levels after taking into account different monetary measures and included: low income (≤$30,000/£15,000), moderate income ($30,000–59,000/£ 15,000–30,000), or high income (≥$60,000/£30,000). Those who did not provide their data on their educational status or annual income were coded as being non-responders and were not included in the analyses.

The SES composite measure combined income and education into a low, moderate, high scale. Participants with low education and low income were categorized as having “low” SES. Those with any combination of moderate or high education and income were deemed to have “high” SES. All other combinations of income and education were categorized as being “moderate” SES. For the main study population (n = 4,988), 21.0% had low SES, 53.6% had moderate SES, and 25.4% had high SES at baseline. SES could not be assessed in 5.9% of the main study population due to missing data in either reported income or education at baseline. Missing income data accounted for the majority of missing values in SES.

### 2.7. Outcome Measures

Cessation outcome measures include: (1) making a quit attempt; (2) quitting among the entire baseline sample; and (3) quitting among those who made a quit attempt (successful quit attempt). All outcome measures are based on self-report and are described below.

Attempts to stop smoking were assessed in the follow-up survey waves among current smokers at baseline by asking: “Have you made any attempts to stop smoking since we last talked with you?” Those responding “yes” were categorized as having made at least one quit attempt between survey waves.

Individual smoking status at time of interview is determined using self-reported smoking status. ‘Current smokers’ were respondents who reported smoking daily, weekly, or monthly at the time of the survey. ‘Former smokers’ were respondents who reported quitting in the time since last contact. This includes respondents who reported quitting: (1) in the last month, (2) 1 to 6 months ago, or (3) 6 to 12 months ago. This was used for two outcomes, quit at follow-up and successful quitting, which includes quitting at follow-up among only those who reportedly made a quit attempt. This measure of “successful quitting” represents the success rate, or the proportion of quit attempts that result in actual cessation.

### 2.8. Statistical Methods

The ITC project is subject to relatively high levels of attrition due to the longitudinal design. Therefore, all analyses have been weighted to adjust for loss to follow-up, using longitudinal survey weights. The weighting techniques and procedures developed and used in the ITC-4 study have been published elsewhere [23]. All statistical analyses were completed using SPSS version 14.0. Multivariate logistic regression analysis was used to examine whether price minimizing behaviors at baseline were predictors of cessation outcomes at follow up. Statistical tests for interaction were performed to examine the joint effects of various price avoidance behaviors and socio-economic status (low, moderate, or high) using multivariate logistic regression modeling. All models presented in this manuscript are adjusted for the covariates described above.

Sub-analyses of long-term cessation outcomes at wave 7 were performed among 3,387 participants who were present at waves 5, 6 and 7, and were current smokers at wave 5 and wave 6. This analysis examines whether long-term use of low or untaxed tobacco sources or tax status changes between waves 5 and 6 were associated with cessation outcomes at follow-up using multivariate logistic regression modeling. Tests for interaction were also performed using the same methods as described above.

## 3. Results

### 3.1. Univariate Analyses and Demographic Characteristics of the Study Population

Baseline characteristics of the study population overall and stratified by country are given in Table 1. Between wave 5 and wave 6, 37.4% reported making a quit attempt and 10.8% were quit at the time of the wave 6 survey. Among the 3 wave cohort (wave 5 to wave 7), 37.5% of current smokers at wave 6 made quit attempts between waves 6 and 7, and 10.2% were quit at the follow-up. The self-reported mean price per cigarette ($USD for year 2006) was lower for all price minimizing behaviors studied compared to full tax/price cigarettes at last purchase (Table 2), validating these measures as being cost-saving behaviors. This pattern was observed among all four countries.

### 3.2. Price Minimizing Behaviors as Predictors of Making a Quit Attempt between Survey Waves

Based on chi-square analysis, for all price minimizing behaviors studied, a significantly smaller proportion of smokers reporting use at baseline made quit attempts at follow-up (Table 3). However, after adjusting for other covariates, purchasing from low/untaxed sources, using discount brands, or using RYO tobacco products at baseline were not predictive of making a quit attempt between baseline and follow-up. Respondents who purchased cartons of cigarettes were approximately 45% less likely to report making quit attempts (OR = 0.55, 95% CI: 0.47–0.65, Table 3) than those who did not purchase cartons. Additionally, combining these measures into “any use” at baseline, respondents who reported at least one price/tax avoidance technique at last purchase were 24% less likely to report quit attempts compared to those who did not use any avoidance behaviors (OR = 0.76, 95% CI: 0.67–0.87). Increasing the number of price minimizing behaviors used resulted in decreased likelihood of making a quit attempt, as respondents who reported using two or more behaviors simultaneously were 31% less likely to report this cessation outcome at follow-up (OR = 0.69, 95% CI: 0.57–0.84). SES did not moderate any of the relationships between the price minimizing behaviors studied and making quit attempts.

### 3.3. Price Minimizing Behaviors as Predictors of Quitting at Follow-up

Based on univariate analyses, use of the various price minimizing behaviors at baseline was associated with a decreased proportion of smokers quitting at follow-up; however, this difference was not statistically significant for use of discount brands or RYO tobacco (Table 3). After adjusting for covariates, respondents who purchased from low and untaxed sources at baseline were approximately 31% less likely to report cessation at follow-up (OR = 0.69, 95% CI: 0.45–1.00). Additionally, respondents who purchased tobacco in cartons were about 33% less likely to be quit at follow-up (OR = 0.67, 95% CI: 0.52–0.87). Similar to the univariate analyses, using discount brands or RYO tobacco was not significantly associated with cessation, although the point estimates are in the predicted direction suggesting use may confer a reduced likelihood of cessation. Based on the composite measure of “any use” of price or tax avoidance behaviors, respondents who used at least one strategy were about 28% less likely to report cessation (OR = 0.72, 95% CI: 0.59–0.88), whereas respondents who engaged in multiple price minimizing behaviors simultaneously (2 or more) were about 41% less likely to quit at follow up (OR = 0.59, 95% CI: 0.42–0.81). No statistically significant interactions were present for any price or tax avoidance behaviors with SES on cessation at follow-up.

### 3.4. Price Minimizing Behaviors as Predictors of Cessation among Those Reporting Quit Attempts

There were no significant associations between use of price minimizing behaviors and successful quit attempts based on univariate analyses (Table 3). After adjusting for covariates, respondents who used discount or generic brand cigarettes were about 25% less likely to report a successful quit attempt (OR = 0.75, 95% CI: 0.59–0.97). Based on the main effect analysis, it appears that purchasing from low and untaxed sources may result in reduced success, but this failed to reach statistical significance. However, a statistically significant interaction was present between using low/untaxed sources and SES for this cessation outcome (p = 0.043). After stratifying by SES, in both the moderate and low SES strata, respondents using low and untaxed sources were about 55% less likely to successfully quit after making an attempt, whereas in the high SES strata, respondents using low and untaxed sources appear to be more likely to successfully quit. However, this relationship was only statistically significant among respondents with moderate SES (Table 3, legend). There were no differences in successful quit attempts among those who did and did not purchase tobacco in cartons or with any use of price or tax avoidance behaviors. However, simultaneous use of two or more price/tax avoidance behaviors was associated with a 32% reduction in successful quit attempts (OR = 0.68, 95% CI: 0.47–0.98). No other statistically significant interactions were present.

### 3.5. Long Term Cessation Associated with Low/Untaxed Source Use among the 3-Wave Cohort

Reported use of low or untaxed sources at waves 5 and at wave 6 was associated with a 57% reduction in making a quit attempt between wave 6 and wave 7 (OR = 0.43, 95% CI: 0.29–0.62; Table 4), compared to those who reported purchasing from a full tax source in both survey waves.

Additionally, participants who reported switching from a full taxed venue at wave 5 to a low or untaxed venue at wave 6 were 32% less likely to report making a quit attempt between waves 6 and 7 (OR = 0.68, 95% CI: 0.46–1.00).

Participants who continued to use low and untaxed sources at waves 5 and 6 were also 47% less likely to report cessation at wave 7 (OR = 0.53, 95% CI: 0.29–0.97). Those who reported switching from full taxed sources to low or untaxed sources also appear to be less likely to report cessation at wave 7, although this failed to reach statistical significance. Conversely, respondents who reported switching from low or untaxed sources to full taxed sources appear to be more likely to quit smoking at wave 7, although again this was marginally non-significant. No significant interactions were present for differing purchase locations and SES on making a quit attempt or cessation at wave 7.

## 4. Discussion

Data from this study indicate that a sizeable percentage of smokers from four high-income countries engage in behaviors aimed at obtaining lower priced cigarettes, by purchasing cheaper tobacco brands, utilizing low or untaxed tobacco retail outlets, using self-made (RYO) tobacco products, or purchasing tobacco in bulk (cartons). These price minimizing behaviors may decrease the public health benefits that are had from increasing cigarette prices through taxation, as engaging in price minimizing behaviors were associated with decreased likelihood of both making quit attempts and reporting cessation at follow-up.

Overall, there does not appear to be a statistically significant interaction between socio-economic status (SES) and purchasing patterns on cessation outcomes. Although respondents with low SES differentially use varying types of price minimizing behaviors [24] the findings presented here suggest that the relationship between using price minimizing behaviors and cessation outcomes does not vary by SES. Therefore, tobacco control policies aimed at decreasing use or eliminate the price differentials between lower priced and full priced cigarettes should have equivalent effects on all segments of the market (*i.e.*, without regard to SES groups).

For all price minimizing behaviors examined, it appears that use inhibits cessation outcomes. However, it is important to examine and understand how each price minimizing behavior affects the specific cessation outcomes.

This study indicated that purchasing from low or untaxed sources overall has a negative effect on cessation, but does not appear to inhibit respondents from making quit attempts. However, the opposite results were obtained using previous ITC data from waves 1 and 2 [7]. This study, which also assessed use of low or untaxed sources on cessation outcomes, found that use was associated with making fewer quit attempts but use was not associated with cessation [7]. One such explanation for this discrepancy over time could be due to the relatively small proportion of smokers who purchased from low or untaxed sources in previous waves as use was only reported by less than 1% in AU, 3.1% in CA, 4.8% in the US, and 15.3% in the UK.

Use of discount or generic brand cigarettes at last purchase was associated with a reduced cessation success rate among those who attempted to quit. Similar to using low or untaxed sources, results of this study showed that using discount or generic brand cigarettes did not inhibit smokers from making quit attempts. Although not statistically significant, the point estimate associated with overall cessation in the current study was in the predicted direction, suggesting that use of discount brands may also reduce overall cessation. A previous study of US adults by Cummings, et al (1997) concurs, finding that use of discount or generic brands was associated with a 21% reduction in cessation at follow-up [23]. However, this study was performed when discount and generic brands were first introduced into the US market, resulting in a large influx of highly ubiquitous, cheaper tobacco products. As evidence of this, during this study’s follow-up period, 23% of respondents had switched from a premium brand to a discount brand [23]. This in turn may have contributed to the observed reduction in smoking cessation. The lack of statistical association in the current study may therefore be a result of the timing of the study and a much shorter follow-up period.

Purchasing cartons was associated with reduced likelihood of both making quit attempts and cessation even after adjusting for smoking dependence and socio-economic differences. There was no significant effect observed for successful cessation among those who tried to quit. This suggests that purchasing tobacco in cartons has a much larger impact on initially inhibiting smokers from making quit attempts, however among those trying to quit, use does not necessarily affect their success rate. This price minimizing behavior essentially increases the supply of available tobacco, which may partially explain why rates of cessation and quit attempts are lower. For example, results from a focus group study of Mexican adults [28] found that many participants reported purchasing single cigarettes as a method of quitting, cutting down, or keeping from smoking too many cigarettes. Having a constant supply of tobacco available was associated with smoking cigarettes at a higher frequency in this group. Additionally, data from these focus groups showed that single cigarettes cost more money and were less available [28]. In contrast, purchasing tobacco in bulk decreases the cost per cigarette and increases individual smokers’ tobacco availability, possibly contributing to higher consumption and lower rates of cessation outcomes.

Use of any price minimizing strategy is associated with reduced quit attempts and overall cessation. Among those who made a quit attempt, there also appears to be a reduction in successful quitting with use of any price or tax avoidance, however this marginally insignificant. This is consistent with previous literature which suggests that individual price minimizing behaviors have been associated with decreased likelihood of making quit attempts [7,29]. Additionally, respondents who reported using multiple price minimizing strategies simultaneously were less likely to report making quit attempts and were even less likely to report cessation and successful quit attempts at follow-up. This implies that smokers who are willing to work harder to maintain their “drug supply” are less likely to both try to quit and to maintain abstinence. Additionally, these results suggest that high levels of price and tax avoidance broadly, rather than any particular type of behavior, are important inhibitors of cessation and quit attempt success. Therefore, price minimizing strategies in general represents a greater public health threat compared to any individual source alone.

In the current study, measures of price minimizing behaviors at last purchase are used as proxy measures for usual purchasing behaviors. Although last and usual behaviors may not be the same, these two measures were highly correlated in this study population at baseline. Usual and last purchase location was the same in approximately 90% of smokers. Additionally, nearly 95% of last brands purchased were the same as the usual brand smoked (analysis not shown). This suggests that the associations between last purchase and cessation measures from this paper are generalizable to usual purchasing patterns as well.

In the current study, consistent use of low and untaxed sources at waves 5 and 6 was significantly associated with decreased likelihood of trying to quit and with decreased long term cessation compared to full tax purchasers at both survey waves. This is consistent with previous work by Hyland, *et al.*, who found that Upstate New York respondents who typically purchased cigarettes from an Indian Reservation were about 55% less likely to report making a quit attempt and 40% less likely to have quit at follow up, compared to participants who reported usually purchasing their cigarettes from other sources [29]. Therefore, tobacco control policies which eliminate these sources and institute equal tax or price increases across all sections of the tobacco market may have a greater impact on consistent users of price and tax avoidance techniques, as this group may no longer be able to afford their usual smoking behaviors.

Although price and tax increases are accepted policy interventions for reducing smoking [1–5], the resulting higher prices could also increase use of price minimizing behaviors, possibly attenuating the effect of the tax itself. For example, after a large tax increase in New York City, although smoking rates declined, the proportion of cigarettes reportedly purchased outside of the city limits increased by 89%. Most of these alternative purchases were from lower taxed jurisdictions such as from New York State (outside of the city, tax jurisdiction), in a different state, over the internet, from another person, or from an Indian Reservation [4]. However, smokers who already engage in multiple price minimizing behaviors should be more likely to quit in the presence of a significant price increase due to the limited number of price minimization strategies available. This topic area requires further research, and we plan to explore this in relation to large one-off price increases from the USA in 2009 and in Australia in 2010 using ITC data.

The relationship between use of price minimizing behaviors and cessation outcomes could also be related to other individualized smoking characteristics such as levels of dependence or lack of motivation to quit. For example, smokers who continue to use tobacco products stripped of much of their ancillary (value-added) appeal or those who purchase tobacco products by the carton, may be more addicted overall. Therefore, it is possible that use of these products is a sign of higher dependence, rather than a cause of reduced cessation success. However, we attempted to adjust for dependency using a measure which combined smokers’ reported cigarettes per day and time to first cigarette upon waking in all multivariate analyses. Future research may be needed to understand the relationship between these measures with use of price minimizing behaviors on cessation outcomes.

According to our model of compensatory behaviors, use of price minimizing behaviors is one choice in the wake of a price or tax increase. However, it is possible that use of price minimizing behaviors is also driven by high tobacco prices. Given the known relationship between high prices and smoking cessation, high tobacco prices could confound the relationship between use of price minimizing behaviors and cessation. Although not presented in this manuscript, after adjusting for an exogenous price variable, the relationship between each price minimizing behaviors and cessation behaviors were still present, suggesting that price did not in fact confound these relationship.

Data from this manuscript indicate that there are no significant differences in cessation outcomes by socio-economic status for select purchasing patterns. There may be several explanations for this lack of statistical interaction. Firstly, as the data suggest, SES may not impact the associations between purchasing patterns and cessation outcomes, and these relationships may be due to differences in dependence or other factors related to the individual. This scenario would benefit public health policy makers, in that new policy initiatives aimed at price and purchasing behaviors would have nearly the same affect on all members of the population, regardless of social class. However, analyses in this manuscript relied on a SES composite measure that was developed using self-reported income and education data. Therefore, it is possible that the composite measure is not completely representative of each participant’s true SES. More detailed SES measures may be needed to fully understand the relationship between price minimizing behaviors and cessation across different stratum of SES.

Several policy interventions can be implemented that specifically address the availability of cheaper tobacco products. Examples include those policies which are called for or based on aims set forth in the Framework Convention on Tobacco Control (FCTC), such as those to initiate large scale efforts to eliminate illicit trade of tobacco or those banning duty-free sales. Both of these policy interventions aim to reduce the supply of lower priced tobacco products [30]. In addition, the tobacco industry often targets specific population subgroups with price promotion materials, which also serve to stimulate in-store sales [5,31]. Comprehensive marketing bans, including bans on price promotions, can help to reduce the availability of cheaper tobacco products. Setting minimum price laws which prohibit promotional incentives such as buy-downs or other programs may also effectively increase cigarette prices at the retail level, while also reducing use of promotional materials [31]. Recently, the United States Government passed the PACT Act which prohibits common courier services, including the United States Postal Service (USPS) from distributing cigarettes and smokeless tobacco products which may not have proper taxes assessed [32]. However, other lower priced venues still provide cheaper alternatives which must be addressed through additional policy interventions.

## 5. Limitations

Although this study has several strengths including a robust sample size of international participants, detailed purchasing behavior information, and longitudinal data that has been validated for use in this international population, there are also limitations.

Firstly, the ITC study relies on self reported data. However, this study has been pilot tested and validated for use in all four countries using self-reported responses. Another limitation of the ITC study is the attrition rates between survey waves. Among all smokers interviewed at baseline, approximately 29% were lost to follow-up one year later. After examining baseline characteristics between lost and retained respondents, a greater proportion of those lost to follow-up were younger, from minority racial or ethnic groups, and had slightly lower income and education. Although differences existed, the magnitude of difference was not substantial, and all analyses were weighted to be representative of the population within each country [24]. Therefore, it is unlikely that attrition has influenced the results presented here. Supplementary analyses found that tobacco prices did not confound the relationship between use of price minimizing behaviors and cessation. Although self-reported and exogenous measures of tobacco price were significantly correlated, neither measures of tobacco price may be fully sufficient to examining this relationship. Further research is needed to develop a more representative price measure that can be used to elucidate this relationship.

Analyses in this manuscript also relied on a composite SES measure that was developed using self-reported categorical data on income and education. Therefore, it is possible that the composite measure is not truly representative of each participant’s SES status. In addition, respondents with missing data in the SES composite measure were not included in the analysis and could introduce bias. However, missing data were evenly distributed between persons who did and did not engage in price minimizing behaviors. Therefore, any bias introduced would most likely be non-differential, and point estimated would be biased toward the null. More detailed SES measures may be needed to fully understand the relationship of SES and cessation with use of price minimizing behaviors.

## 6. Conclusions

The estimates presented in this paper indicate that the availability of lower priced cigarette alternatives may attenuate public health efforts aimed at reducing smoking prevalence through price and tax increases. The relatively high availability of lower cost tobacco products may result in alternative options to cessation in people who may have otherwise quit in the wake of increasing cigarette prices. This paper also shows that high use rates of price minimizing behaviors (simultaneous use of sources) was associated with an even lower likelihood of cessation outcomes compared to only using one source. Additionally, long term use of low and untaxed sources resulted in consistently lower rates of cessation indicators at long-term follow-up.

However, the results of this paper suggest that there is no interaction present between price minimizing behaviors and SES on cessation outcomes. Therefore, policies aimed at eliminating or reducing the price differentials or availability of less expensive alternatives should be equally effective among individuals with varying socio-economic levels.

## Figures and Tables

**Table 1.** Demographic Characteristics of participants who were current smokers present in waves 5 (main/recruitment) and at wave 6 follow-up, overall (n=4988) and stratified by country (CA: n = 1245; US: n = 1130; UK: n = 1263; AU: n = 1350) (n, % [unweighted]).

**Characteristic**	**Overall (n = 4,988)**	**CA (n = 1,245)**	**US (n = 1,130)**	**UK (n = 1,263)**	**AU (n = 1,350)**
**Country**					
Canada	1,245 (25.0)				
United States	1,130 (22.7)				
United Kingdom	1,263 (25.3)				
Australia	1,350 (27.1)				

**Sex**					
Female	2,867 (57.5)	723 (58.1)	680 (60.2)	715 (56.6)	749 (55.5)
Male	2,121 (42.5)	522 (41.9)	450 (53.9)	548 (43.4)	601 (44.5)

**Age at Wave 5**					
18–39	1,272 (25.5)	318 (25.5)	208 (18.4)	315 (24.9)	431 (31.9)
40–54	2,069 (41.5)	542 (43.5)	487 (43.1)	482 (38.2)	558 (41.3)
55+	1,647 (33.0)	385 (30.9)	435 (38.5)	466 (36.9)	361 (26.7)

**Ethnicity**					
White	4,509 (90.5)	1,144 (91.9)	955 (84.8)	1,203 (95.5)	1,207 (89.4)
Non-White	472 (9.5)	101 (8.1)	171 (15.2)	57 (4.5)	143 (10.6)

**Income**					
Low	1,516 (32.5)	315 (27.1)	385 (35.7)	413 (35.6)	403 (31.7)
Moderate	1,677 (35.9)	455 (39.1)	405 (37.6)	404 (34.8)	413 (32.5)
High	1,478 (31.6)	393 (33.8)	288 (26.7)	343 (29.6)	454 (35.7)

**Education**					
Low	2,595 (52.0)	557 (44.8)	472 (41.8)	743 (59.4)	823 (61.0)
Moderate	1,543 (31.0)	463 (37.2)	432 (38.3)	334 (26.7)	314 (23.3)
High	832 (16.7)	223 (17.9)	224 (19.9)	173 (13.8)	212 (15.7)

**SES**					
High	1,182 (25.4)	349 (30.0)	279 (27.6)	254 (22.1)	282 (22.2)
Moderate	2,495 (53.6)	626 (53.9)	572 (53.1)	594 (51.7)	703 (55.4)
Low	980 (21.0)	187 (16.1)	208 (19.3)	301 (26.2)	284 (22.4)

**CPD**					
mean (SD)	17.29 (10.0)	16.25 (9.1)	18.55 (10.8)	16.34 (9.4)	18.10 (10.4)

**Minutes to 1st cig**					
mean (SD)	66.62 (135.6)	58.66 (121.7)	58.23 (121.7)	68.74 (131.9)	78.99 (159.3)

**HSI**					
mean (SD)	2.69 (1.54)	2.7 (1.51)	2.82 (1.57)	2.53 (1.45)	2.73 (1.61)

**Smoking Status (W5)**					
Daily	4,725 (94.7)	1,179 (94.7)	1,079 (95.5)	1,203 (95.2)	1,264 (93.6)
Weekly	211 (4.2)	50 (4.0)	42 (3.7)	53 (4.2)	66 (4.9)
Monthly	52 (1.0)	16 (1.3)	9 (0.8)	7 (0.6)	20 (1.5)

**Smoking Status (W6)**					
Current	4,456 (89.3)	1,129 (90.7)	1,020 (90.3)	1,103 (87.3)	1,204 (89.2)
Quit	532 (10.7)	116 (9.3)	110 (9.7)	160 (12.1)	146 (10.8)

All by country comparisons are statistically significant at p < 0.05 level based on chi square analyses. CPD = Cigarettes per day (baseline). SD = Standard Deviation. HSI = Heaviness of Smoking Index (baseline); composite measure of CPD and Minutes to 1st cigarette after waking, range from 0 to 6 with 6 being more addicted. In all subsequent models, only HSI will be added into the model with values ranging from 1 to 6.

**Table 2.** Mean price per cigarette for various price/tax avoidance behaviors, stratified by country.

**Purchasing Behavior at Baseline**	**Average Cost Per Cigarette Mean (Standard Deviation)**			
	**CA**	**US**	**UK**	**AU**
**Last Purchase Location:**				
Full Tax Source	$0.30 (0.063)	$0.18 (0.065)	$0.46 (0.057)	$0.31 (0.040)
Low/untaxed Source	$0.12 (0.067)	$0.13 (0.053)	$0.22 (0.084)	$0.19 (0.113)

**Tobacco Type:**				
Premium	$0.32 (0.060)	$0.19 (0.055)	$0.45 (0.118)	$0.32 (0.044)
Discount	$0.25 (0.071)	$0.14 (0.065)	$0.43 (0.065)	$0.28 (0.044)

**Purchase by Carton:**				
No	$0.30 (0.064)	$0.20 (0.061)	$0.46 (0.053)	$0.21 (0.39)
Yes	$0.23 (0.090)	$0.14 (0.049)	$0.34 (0.113)	$0.28 (0.051)

**Any Use:**				
No	$0.33 (0.048)	$0.21 (0.053)	$0.50 (0.049)	$0.33 (0.034)
Yes	$0.26 (0.071)	$0.15 (0.056)	$0.41 (0.096)	$0.28 (0.038)

**Varying Use:**				
0 sources	$0.33 (0.048)	$0.21 (0.053)	$0.50 (0.049)	$0.33 (0.034)
1 source	$0.28 (0.051)	$0.17 (0.053)	$0.44 (0.048)	$0.29 (0.027)
2+ sources	$0.20 (0.081)	$0.13 (0.051)	$0.30 (0.124)	$0.27 (0.053)

Average cost per cigarette is based on self-reported price at last purchase after adjusting for inflation and currency (excludes RYO tobacco). Prices are given in US dollars for the year 2006. Analysis of price per cigarette is only completed among values which are within three standard deviations of the mean (range: $0.00 to $0.69).

**Table 3.** Purchasing Behaviors at baseline as predictors of Cessation Outcomes at 1 year follow-up.

	**Overall Behavior Use**		**Univariate Analysis ^a^: Percent Reporting Cessation Outcome by Behavior**			**Multivariate Analysis ^b^**		

**Behavior at Last Purchase**	**N**	**%**	**Make Quit Attempt**	**Overall Quit at Follow-up**	**Successful Quit Attempt**	**Make a quit attempt**	**Quit at follow-up**	**Successful Quit Attempt**
**Use Discount Brand or RYO Tobacco**								
No	2,279	8.7%	**38.6%**	11.8%	30.5%	1.00	1.00	1.00
Use Discounts	1,846	34.9%	**37.4%**	9.7%	26.0%	1.07 (0.93–1.23)	0.86 (0.69–1.07)	**0.75 (0.59–0.97)**
Use RYO	756	16.5%	**33.6%**	10.0%	29.9%	0.94 (0.78–1.13)	0.76 (0.57–1.02)	0.74 (0.53–1.04)
*p based on chi square*			*p = 0.038*	*p = 0.083*	*p = 0.144*			
*p for interaction with SES*						*p = 0.186*	*p = 0.211*	*p = 0.096*

**Use Low or Untaxed Source**								
No	4,455	90.7%	**38.1%**	**11.2%**	29.3%	1.00	1.00	1.00
Yes	502	9.3%	**30.6%**	**7.7%**	25.0%	0.87 (0.70–1.09)	**0.69 (0.45–1.00)**	0.72 (0.47–1.11)
*p based on chi square*			*p = 0.002*	*p = 0.022*	*p = 0.332*			
*p for interaction with SES*						*p = 0.381*	*p = 0.218*	p = 0.043**

Purchase tobacco by Carton (last)*								
No	2,779	69.7%	**42.8%**	**12.7%**	29.7%	1.00	1.00	1.00
Yes	1,467	30.3%	**28.0%**	**7.5%**	26.8%	**0.55 (0.47–0.65)**	**0.67 (0.52–0.87)**	1.02 (0.75–1.37)
*p based on chi square*			*p < 0.001*	*p < 0.001*	*p = 0.320*			
*p for interaction with SES*						*p = 0.672*	*p = 0.460*	*p = 0.705*

**Use at least one strategy**								
No	1,496	34.5%	**43.5%**	**13.4%**	30.6%	1.00	1.00	1.00
Yes	3,340	65.5%	**34.5%**	**9.6%**	27.9%	**0.76 (0.67–0.87)**	**0.72 (0.59–0.88)**	0.79 (0.63–1.01)
*p based on chi square*			*p < 0.001*	*p < 0.001*	*p = 0.218*			
*p for interaction with SES*						*p = 0.232*	*p = 0.837*	*p = 0.926*

**Number of Strategies Used**								
0	1,496	34.5%	**43.5%**	**13.4%**	30.6%	1.00	1.00	1.00
1	2,377	48.4%	**36.3%**	**10.4%**	28.8%	**0.78 (0.68–0.90)**	**0.75 (0.61–0.93)**	0.83 (0.65–1.06)
2+	963	17.1%	**31.0%**	**8.0%**	43.9%	**0.69 (0.57–0.84)**	**0.59 (0.42–0.81)**	**0.68** (0.47–0.98)
*p based on chi square*			*p < 0.001*	*p < 0.001*	*p = 0.265*			
*p for interaction with SES*						*p = 0.460*	*p = 0.575*	*p = 0.713*

* Purchasing by carton excludes respondents who purchased RYO Tobacco.

a Univariate Analyses: Weighted percentages; p values passed on chi square analysis. **Bolded entries** are statistically significant at p < 0.05 level.

b Multivariate Analyses: Each row represents a separate multivariate logistic regression model assessing whether the purchasing behavior of interest was predictive of cessation indicators.

at follow-up. All OR’s are adjusted for: SES, country, age, sex, ethnicity, HSI.

** There was a significant interaction by SES for those using low/untaxed sources and making a successful quit attempt. Stratified analysis by SES: High SES (OR = 1.555, 95% CI: 0.817–2.959) ***Moderate (OR = 0.445 95% CI: 0.225–0.880)*** Low SES (OR = 0.440 95% CI: 0.135–1.439)

**Table 4.** Changing location of last purchase between waves 5 and 6 as predictors of Long Term Cessation Outcomes at Wave 7 follow-up.

**Purchase Location at**		**Long Term Cessation Outcome at Wave 7**			
			**Quit Attempt** (n = 3,117)		**Cessation** (n = 3,117)

**Wave 5**	**Wave 6**	N	OR (95% CI)	N	OR (95% CI)

**Full Tax Source**	**Full Tax Source**	2,620	1.00	2,620	1.00
**Full Tax Source**	**Low/Untaxed Source**	157	**0.68 (0.46–1.00)**	157	0.60 (0.31–1.17)
**Low/Untaxed Source**	**Full Tax Source**	113	1.12 (0.76–1.64)	113	1.63 (0.99–2.69)
**Low/Untaxed Source**	**Low/Untaxed Source**	227	**0.43 (0.29–0.62)**	227	**0.53 (0.29–0.97)**

**Bolded** entries represent significant associations at p < 0.05 level; All models adjusted for: SES, Country, Age, Sex, Ethnicity, and Heaviness of Smoking Index. Quit attempt and Cessation between wave 6 and 7 were assessed among participants with complete data who were current smokers at both waves 5 and 6. *P for interaction between tax status change and SES on making a quit attempt at wave 6: p = 0.935. P for interaction between tax status change and SES on making a quit attempt at wave 6: p = 0.763.*
